# Testis transcriptome analysis in male infertility: new insight on the pathogenesis of oligo-azoospermia in cases with and without AZFc microdeletion

**DOI:** 10.1186/1471-2164-11-401

**Published:** 2010-06-24

**Authors:** Valentina Gatta, Florina Raicu, Alberto Ferlin, Ivana Antonucci, Anna Paola Scioletti, Andrea Garolla, Giandomenico Palka, Carlo Foresta, Liborio Stuppia

**Affiliations:** 1 Department of Biomedical Sciences, "G. d'Annunzio" University, Chieti-Pescara, Italy; 2 Aging Center Research, "G. d'Annunzio" Foundation, Chieti-Pescara, Italy; 3 Carol Davila University of Medicine and Pharmacy, Genetics Chair, Bucharest, Romania; 4 Department of Clinical Sciences and Imaging, "G. d'Annunzio" University, Chieti-Pescara, Italy; 5 Centre for Male Gamete Cryopreservation, Department of Histology, Microbiology, and Medical Biotechnologies, University of Padova, Italy; 6 Human Genetics Division, "Spirito Santo" Hospital, Pescara, Italy; 7 IGM-CNR c/o IOR, Bologna, Italy

## Abstract

**Background:**

About 10% of cases of male infertility are due to the presence of microdeletions within the long arm of the Y chromosome (Yq). Despite the large literature covering this critical issue, very little is known about the pathogenic mechanism leading to spermatogenesis disruption in patients carrying these microdeletions. In order to identify the presence of specific molecular pathways leading to spermatogenic damage, testicular gene expression profiling was carried out by employing a microarray assay in 16 patients carrying an AZFc microdeletion or affected by idiopathic infertility. Hierarchical clustering was performed pooling the data set from 26 experiments (16 patients, 10 replicates).

**Results:**

An intriguing and unexpected finding is that all the samples showing the AZFc deletion cluster together irrespectively of their testicular phenotypes. This cluster, including also four patients affected by idiopathic infertility, showed a downregulation of several genes related to spermatogenesis that are mainly involved in testicular mRNA storage. Interestingly, the four idiopathic patients present in the cluster showed no testicular expression of *DAZ *despite the absence of AZFc deletion in the peripheral blood.

**Conclusions:**

Our expression profiles analysis indicates that several forms of infertility can be triggered by a common pathogenic mechanism that is likely related to alterations in testicular mRNA storage. Our data suggest that a lack of testicular DAZ gene expression may be the trigger of such mechanism. Furthermore, the presence of AZFc deletions in mosaic or the loss of function of AZFc genes in absence of Yq deletion can perhaps explain these findings. Finally, based on our data, it is intriguing to hypothesize that *DAZ *gene dysfunctions can account for a larger number of previously thought "idiopathic" infertility cases and investigation of such testicular gene dysfunction can be important to reveal the molecular determinant of infertility than are undetected when only testing Yq deletions in peripheral blood.

## Background

Microdeletions of the Y chromosome long arm (Yq) represent the main molecular determinants of male infertility and account for about 10% of cases of non obstructive azoospermia or severe hypospermatogenesis [1-5]. Yq microdeletions involve three Azoospermia Factors (AZF) loci, AZFa, AZFb and AZFc [6] and remove many genes likely involved in male germ cell development and maintenance [7]. The most frequent deletion of the Y chromosome (AZFc, b2/b4) spans 3.5 Mb and eliminates 21 genes and transcription units of the AZFc region. Among these, the main candidate for spermatogenesis failure is the Deleted in Azoospermia (DAZ) gene, a testis specific gene present in four copies within AZFc and encoding for an RNA binding protein [6,7]. The AZFc deletion has been associated with wide range of phenotypes ranging from the complete absence of germ cells in the testes (Sertoli cell only syndrome, SCOS) to a relevant reduction of germ cells still including mature sperm (severe hypospermatogenesis, HS), to a maturation spermatogenesis arrest. Despite the large number of studies investigating the prevalence and the molecular basis of these rearrangements, the testicular gene expression of patients carrying Yq deletions is still largely unknown and so are the molecular mechanisms leading to spermatogenesis disruption.

In recent years, expression profiling of human testis has been widely used for the identification of genes that are involved in different key steps of testis development and function [8-10]. However, at the moment, not a single study has investigated the testicular transcriptome of patients who carry Yq microdeletions.

In this study, we analyzed testis expression profiles of 16 infertile patients with different testicular phenotypes, including men carrying AZFc deletions as well as subjects with idiopathic infertility.

## Results

**Figure 1:**
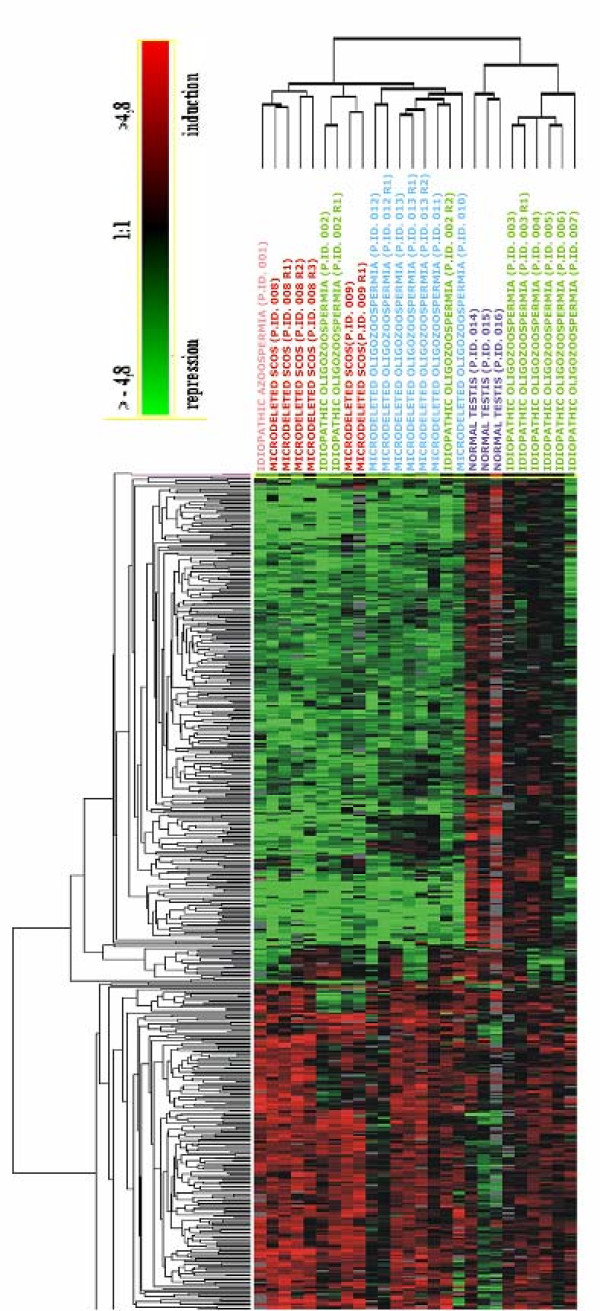
**Hierarchical clustering showing a similar expression profile in all patients with AZFc deletions and in four patients with idiopathic infertility**. In the figure, all the relevant genes are grouped according to their expression values shown as log ratios (ratios are: gene expression value of the pathological testis divided by the value of a normal testis and values of the three samples with normal spermatogenesis divided by the home-made RNA universal). Each row corresponds to one gene, each column to different 26 microarray experiments. The quantitative changes in gene expression across all the samples are represented in color: red indicates overexpressed genes, and green indicates downregulated genes. Black bars indicate no changes in expression. Missing data points are represented as gray bars. Different colors of top labels indicate different testis phenotypes. (Pink= idiopathic SCOS; red = AZFc microdeletion with SCOS phenotype; green = idiopathic HS; blue = AZFc microdeletion with HS phenotype; purple = normal testis; R= replica).

In order to discriminate the genes specifically involved in the different pathological phenotypes, we performed a hierarchical clustering of the data set originated from 26 experiments (16 patients, 10 technical replicates) (Table 1). All the AZFc samples clustered together independently from their testis phenotype (SCOS or HS) (Figure 1). The same cluster also contained 4 patients without Yq deletions (3 affected by idiopathic HS and one by idiopathic SCOS). The remaining three patients without Yq deletions showed a different gene expression profile which was not clustered together with AZFc deleted patients. All the testes with normal spermatogenesis clustered appropriately into their group. The cluster containing AZFc-deleted samples was characterized by the presence of 490 transcripts showing at least a 1.7-fold change in expression as compared to testis with normal spermatogenesis (Additional file 1 and 2). Semiquantitative RT-PCR, carried out on selected genes (*ACTL7B, LDHC, ODF 1, TSSK2, PRM2, TNP1*), confirmed microarray results (additional file 3).

**Table 1:** Testis phenotype of the investigated patients and number of micorarray experiments.

Patients classification	Semen analysis	Testis histology (Johnsen score)	Patient ID	Microarray experiments
Idiopathic SCOS	Azoospermia	SCOS (2)	1	1

Idiopathic HS	≤ 1 × 10^6^/ml	Severe HS (8)	2	3
			3	2
			4	1
			5	1
			6	1
			7	1

AZFc microdeletion SCOS	Azoospermia	SCOS (2)	8	4
			9	2

AZFc microdeletion HS	≤ 1 × 10^6^/ml	Severe HS (8)	10	1
			11	1
			12	2
			13	3

Obstructive azoospermia	Azoospermia	Normal spermatogenesis (10)	14	1
			15	1
			16	1
**Total microarray experiments**				26

**Figure 2:**
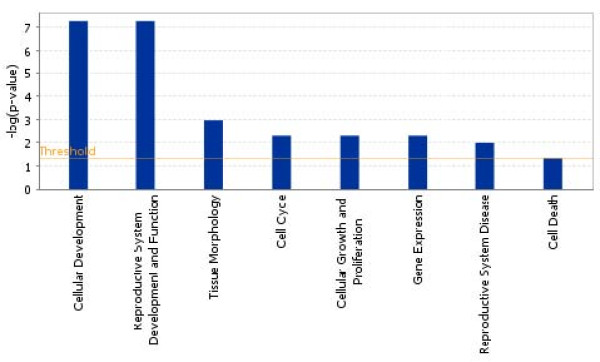
**Histogram showing the main biological functions of downregulated genes present in the cluster of patients with AZFc deletion**.

Ingenuity Pathway Analysis (IPA) was carried out to investigate the main functions played by genes that were found to be downregulated (n. = 331, additional file 1) and showed that "Cellular development" and "Reproductive system development and function" were the main functions correlated with these genes (Figure 2). These functional categories contained several genes playing a role in spermatogenesis, fertilization, and determination of the testicular mass, some of which are involved in human and murine male infertility (*DDX25 *and *FKBP6*) [11] (*SPAG6*) [12] (Table 2).

**Table 2:** Dowregulated genes in the cluster containing AZFc deleted samples as classified on the basis of their main biological functions.

General biological function	Specific biological function	Downregulated genes in AZFc deleted patients cluster
*Cellular development*	Spermatogenesis	AFF4, CCT6B, CRISP2, DDX25, FKBP6, KIF6, PRM2, SPAG6, TNP1, TSGA10

*Reproductive system development and function*	Fertilization	CLGN, DAZ1, TNP1, PCSK4
	Mass of testis	ARL4A, PTDSS2

**Figure 3:**
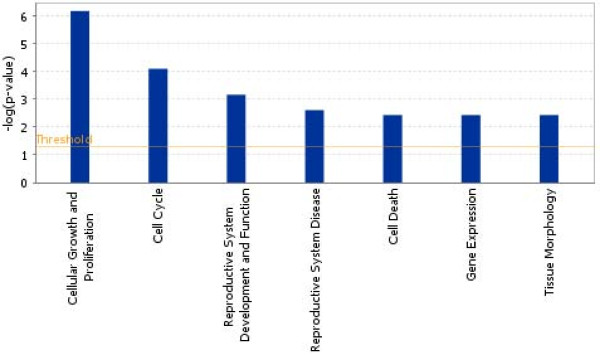
**Histogram showing the main biological functions of upregulated genes present in the cluster of patients with AZFc deletion**.

When we analyzed the upregulated genes (n = 159, additional file 2), IPA showed that "Cellular growth and proliferation" and "Cell death" were the functions to be involved, whereas "Reproductive system development and function" was less represented (Figure 3, Table 3).

**Table 3:** Upregulated genes in the cluster containing AZFc deleted samples as classified on the basis of their main biological functions.

General biological function	Specific biological function	Upregulated genes in AZFc deleted patients cluster
*Cellular growth and proliferation*	Proliferation of eucariothic cell	A2M, ADAMTS1, AIF1, ANGPTL1, CEBPD, COL4A1, DCN, GSTM1, HIF1A, HMGB1, ICMT, IL6ST, KIF3A, LY96, NKX3-1, PLAU, PTK2, S100B, SOD2, SPTBN1, TPT1

*Reproductive system development and function*	Reproductive process of mammalia	DDX3Y (DBY), IL6ST, PLAU, UTY

*Cell death*	apoptosis of eukaryotic cells	A2M, ANGPTL1, C7, CEBPD, DCN, HIF1A, HMGB1, ICMT, IL6ST, KIF3A, PLAU, PTK2, SOD2, TPT1, YES1

**Figure 4:**
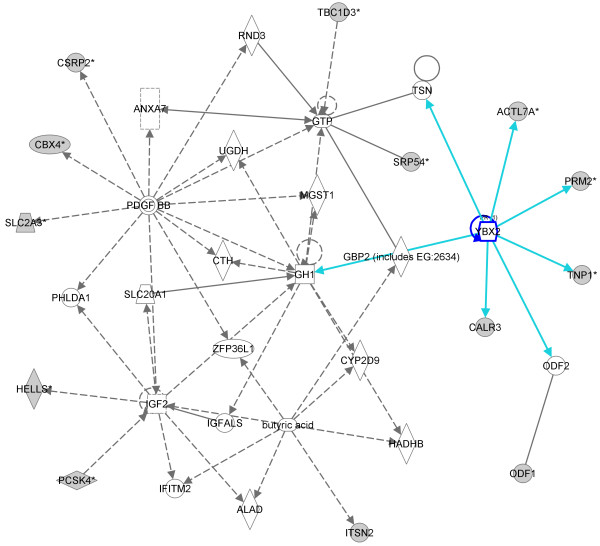
**IPA-originated network of downregulated genes found in the cluster of patients with AZFc deletion**. Network centered on YBX2 gene. The grey shading indicates genes that show decreased expression in the pathological testis when compared with normal testis samples.

**Figure 5:**
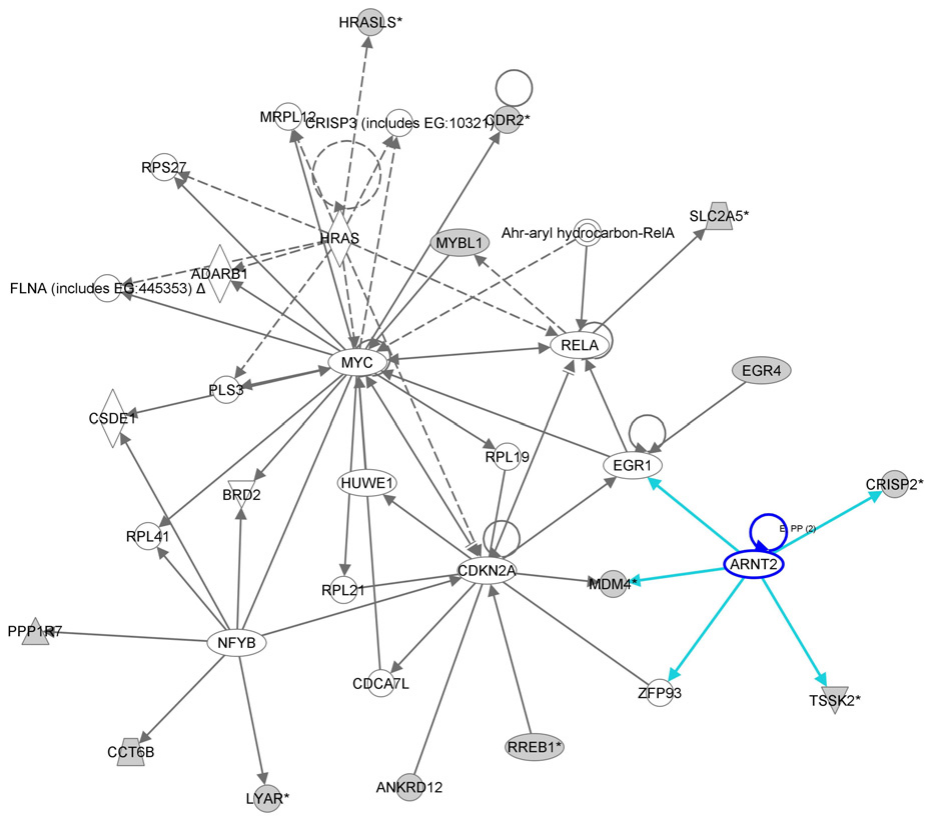
**IPA-originated network involving of upregulated genes found in the cluster of patients with AZFc deletion**. Network centered on ARNT2 gene. The grey shading indicates genes that show increased expression in the pathological testis when compared with normal testis samples.

Network analysis of the dowregulated genes that are linked with spermatogenesis showed an interesting network centered on the *YBX2 *(*MSY2*) gene, a member of the Y-box gene family that is selectively expressed in male and female germ cells (Figure 4) [13]. We also found an additional network that is centered on the *ARNT2 *gene and contains other genes critically involved in the spermatogenetic process (*CRISP2, TSSK2, MDM4 *and *EGR4*) (Figure 5).

A specific analysis of the DAZ gene signal in the samples represented in the cluster showed that *DAZ *was not expressed in all the four patients who were not carrying the AZFc deletion (Table 4). These results were confirmed by a RT-PCR analysis carried out as previously described [14]. The same kind of analysis was carried out also for CDY1 and VCY2 genes, mapped in multiple copies within AZFc locus close to the DAZ gene cluster [7]. This analysis evidenced CDY1 expression in patients with idiopathic infertility but not in those with AZFc deletion. On the other hand, an overexpression of VCY2 gene was detected in 4 out of 6 patients with AZFc deletion. A BLAST search using the VCY2 oligonucleotide sequence spotted on the array discovered a 60% homology with the sequence mapped in Yp, corresponding to a portion of the VCY2 exon 7, in addition to the expected 100% homology with VCY2 coding region.

**Table 4:** Expression of the DAZ gene in the investigated patients as evidenced by microarray analysis. + = presence of expression. - = absence of expression.

Patient ID	Phenotype	Present in the cluster	DAZ expression
1	Idiopathic SCOS	Yes	-

2	Idiopathic HS	No	+

3	Idiopathic HS	No	+

4	Idiopathic HS	No	+

5	Idiopathic HS	Yes	-

6	Idiopathic HS	Yes	-

7	Idiopathic HS	Yes	-

8	AZFc deletion SCOS	Yes	-

9	AZFc deletion SCOS	Yes	-

10	AZFc deletion HS	Yes	-

11	AZFc deletion HS	Yes	-

12	AZFc deletion HS	Yes	-

13	AZFc deletion HS	Yes	-

14	Normal spermatogenesis	No	+

15	Normal spermatogenesis	No	+

16	Normal spermatogenesis	No	+

## Discussion

In the present study, we carried out a microarray analysis to investigate testicular gene expression profiles in patients affected by different forms of infertility. Due to the absence or reduced number of germ cells in these patients, a downregulation of genes with prominent or exclusive germ cell expression was expected in all samples, whereas genes expressed mainly in somatic cells were expected to show increased expression. However, the main purpose of this study was to analyze the inter-individual differences among infertile patients, in order to identify specific expression profiles for each different conditions.

Using this approach we found a similar gene expression profile in all the samples originated from individuals carrying the AZFc deletion as well as in four patients with idiopathic infertility. All these case showed a common gene cluster that contained 490 transcripts (331 down- and 159 upregulated genes). Since all HS patients had the same Johnsen score, the difference in the expression profiles of samples present in the cluster and patients outside the cluster cannot be due to a different number of germ cells in their testis, but likely indicates a real difference in the transcription levels of specific genes. Among the downregulated transcripts, we detected several genes involved in spermatogenesis, fertilization, and male infertility. To clarify a potential pathogenic link between the downregulation of these transcripts and spermatogenesis failure, we carried out an IPA network analysis that revealed two interesting networks. The first network is centered on the YBX2 (MSY2) gene, a member of the Y-box gene family that is specifically expressed in male and female germ cells. *YBX2 *marks specific mRNAs for cytoplasmic storage, stabilization, and suppression of translation [13]. RNA storage is a crucial step during spermatogenesis. RNA synthesis terminates during mid-spermiogenesis and stabilization and storage of mRNAs are key for the encoding of proteins needed during the terminal phases of spermatogenesis [15]. Quite surprisingly, network analysis of the cluster of patients carrying AZFc deletions showed that *YBX2 *was not present in the cluster, being not significantly downregulated in the samples within the cluster as compared to the other samples not present in the cluster. However, several genes whose activity is regulated by *YBX2 *were found to be downexpressed in the network. Among these, we found *ODF1, TNP1 *and *PRM2*, all genes that play a key role in the postmeiotic phase of the spermatogenesis. ODF1 encodes for proteins located on the outside of the axoneme in the mid piece and principal piece of the mammalian sperm tail, thereby maintaining the passive elastic structures and elastic recoil of the sperm tail. TNP1 is a spermatid-specific product of the haploid genome which replaces histones and is itself replaced in the mature sperm by the protamines. *PRM2 *encodes for proteins playing a key role in DNA packaging during sperm differentiation. The combined downregulation of all these genes suggests reduced storage of these transcripts during spermatogenesis, via a *YBX2-*independent process as the *YBX2 *transcript was not found within the cluster. A possible intriguing hypothesis is that the loss of function of the *DAZ *gene, the most important gene disrupted by AZFc deletions [16], leads to a pathogenic defective mRNA storage. Substantiating this idea, RNA-binding proteins encoded by *DAZ *are known to be required for the translational regulation of gene expression that is essential for gametogenesis [17,18]. Furthermore, it has been showed that *Dazl*, the murine homologous of *DAZ*, plays a role in the transport of specific mRNAs via a dynein motor complex, and that the *Dazl*-bound mRNAs is stored at specific sites to be available and used for later developmental processes [19]. Therefore we hypothesize that *DAZ *disruption may represent a possible cause of the reduced storage of important testis transcripts encoded in the first steps of the spermatogenesis.

Interestingly, the second network we analyzed is centered on the *ARNT2 *gene and contains several genes involved in the spermatogenesis process (*CRISP2, TSSK2, MDM4 *and *EGR4*). *CRISP2 *is a testis-enriched protein localized to the sperm acrosome and tail. *TSSK2 *encodes a testis-specific serine/threonine kinase and is bound by the human and murine forms of DAZL. EGR4 is a member of the *Egr *family of zinc-finger transcription factors, and regulates genes critically involved in the early stages of meiosis. Thus, it is intriguing to conceive that also *ARNT2 *may play an important role in the pathogenesis of infertility in AZFc deleted patients.

The microarray analysis of samples from patients with AZFc deletion also showed the presence of 159 upregulated transcripts. It is interesting to note that only a few of these genes have been previously linked to infertility. *A2M*, a gene that is encoding for a protease inhibitor, is up-regulated in the testis of the ABP transgenic mouse that shows an impairment of spermatogenesis [20]. Increased expression of *HIF1A*, an hypoxia-inducible transcription factor, is present in the internal spermatic vein of patients with varicocele [21]. Finally, *HMGB1*, encoding for a high mobility group protein, works as a pro-inflammatory and antibacterial factor in human testis [22]. These data suggest that the overexpression of genes related to cell growth and proliferation as well as to apoptosis in the testis of patients with AZFc deletions may represent an unspecific mechanism of reaction to testis damage, rather than the cause of defective spermatogenesis.

An unexpected finding of the present study is that the four patients without AZFc deletion are included in the same cluster containing samples from AZFc deleted patients. In order to verify if the presence of an intact AZFc region in peripheral blood actually correlates with a normal testicular DAZ expression, we focused our attention to the presence/absence of the specific signals for this gene on the array in all cases contained in the cluster. Interestingly, no DAZ signal was detected in any of the four patients without AZFc deletion. Lack of DAZ expression was expected in the samples from patients affected by idiopathic SCOS, due to the absence of germ cells, but it was surprisingly found in the 3 patients affected by idiopathic HS.

The lack of *DAZ *expression in the testis of infertile patients who are not carrying the AZFc deletion in their peripheral blood has been previously reported and interpreted as the result of somatic AZFc rearrangements producing a mosaic in which Yq deletion is present only in testicular cells [14,23]. Our results strongly confirm this finding, corroborating the hypothesis that a portion of patients classified as affected by idiopathic infertility actually lack DAZ gene expression in their testis, thus showing a pathogenic mechanism similar to the one responsible of infertility in AZFc deleted patients. In order to investigate also other AZFc genes, we analyzed the specific spots for CDY1 and VCY2, in order to verify their expression in patients with and without AZFc deletion. As expected, CDY1 was expressed in infertile patients without AZFc deletion independently fromtheir collocation with or without the cluster, but not in patients with AZFc deletion. On the other hand, expression levels showed by VCY2 gene were inconsistent with those of DAZ and CDY1 genes, being this gene overexpressed in 4 out of the 6 patients with AZFc deletion. In order todisclose the cause of this unexpected result, we performed a Blast search using the VCY2 oligonucleotide sequence spotted on the array, discovering a 60% homology with a sequence mapped within Yp, in addition of the expected 100% homology with VCY2 gene. This Yp sequence matched with the VCY2 exon 7, target of the VCY2 probe present in the array. On the other hand, DAZ and CDY1 probes showed homology only with their target genes. This suggests that the VCY2 oligonucleotide on the array is not specific enough to consider the expression levels of this gene in the context of the present study.

## Conclusions

In summary, our study provides the following novel information: i) all patients with AZFc deletions show a similar testis gene expression profile, independently from their SCOS or HS phenotype, suggesting that all AZFc deletions produce a similar transcriptional pattern and that variability in phenotypes is related to environmental or other genetic mechanisms. ii) one half of the patients with idiopathic infertility cluster together with patients with AZFc deletions, suggesting a common pathogenic mechanism. We hypothesize that a crucial link that can explain this phenomenon is the lack of expression of the *DAZ *gene in the testes these cases. A potential alternative explanation may be related to the presence of an AZFc deletion in mosaic and/or the loss of function of AZFc genes in absence of Yq deletion. If our finding will be confirmed on larger series of patients, *DAZ *gene dysfunctions may be proved to account for a larger portion of cases of infertility than one could expect by simply analyzing the prevalence of Yq deletions among these subjects; iii) we found that several genes that are mainly related to the postmeiotic phase of the spermatogenesis and have been previously reported as involved in male infertility are dowregulated in patients with AZFc deletions. Given the crucial role played by RNA storage during spermatogenesis, the loss of function of *DAZ *can translate to a defective mRNA storage in testis with great pathogenic implications. The identification of the molecular mechanisms underlying the spermatogenesis failure in cases characterized by DAZ loss of function will provide useful information for the disclosure of key molecules which could represent in the future the target for personalized drug therapy.

## Methods

### Patients

The study was approved by the local Ethics Committee of the University of Padova and was in accordance with the Helsinki II Declaration. All participants were asked for and provided their informed consent. The study population was selected from a cohort of 1436 men presenting idiopathic non obstructive azoospermia or severe hypospermatogenesis (sperm count <2 million/ml) [5]. Exclusion criteria were: drug consumption, fever in the previous 6 months, seminal infections, varicocele, systemic diseases, previous cryptorchidism or orchitis, presence of anti-sperm auto-antibodies, hypogonadotrophic hypogonadism, abuse of androgenic (anabolic) steroids, treatment with chemotherapeutic agents or radiotherapy, testicular tumors, karyotype abnormalities, androgen receptor, INSL3 and RXFP2 gene mutations [24,25]. Semen analysis was performed according to the World Health Organization guidelines [26] on at least two occasions separated by three months. The diagnosis of azoospermia was established by pellet analysis after semen centrifugation.

The testicular structure and spermatogenic activity was evaluated with a combination of methods to have the most homogeneous sampling. As a screening procedure, bilateral testicular fine needle aspiration (FNA) was performed as previously described [5,27]. Testicular phenotype was classified as follows (1) Sertoli cell only syndrome (SCOS): complete absence of all germ cells in both testes; (2) hypospermatogenesis (HS): quantitative reduction in the number of germs cells with still presence of mature spermatozoa; and (3) maturation arrest (MA): normal presence of germ cells until a definite step of spermatogenesis and lack of germ cells in the later stages.

Patients showing normal karyotype were submitted to molecular testing for Yq microdeletions as previously described (5). Only men carrying the AZFc-b2/b4 deletion were included in the microarray analysis.

The final selected sample comprised 16 patients (pats. 1-16): one patient (pat. 1) had idiopathic SCOS, 6 patients (pats. 2-7) had idiopathic severe HS, 3 patients (pats. 8-10) had SCOS and AZFc-b2/b4 deletion, and 3 patients (pats. 11-13) had severe HS and AZFc-b2/b4 deletion. Three men with obstructive azoospermia and normal spermatogenesis were used as controls (pats. 14-16) (Tab. 1).

### Testicular histology

Selected subjects underwent bilateral testicular biopsy to clearly assess testicular histology and provide RNA for microarray experiments. At least three 3-5 mm fragments per testis were excised, processed for histological analysis, and at least one sample per testis was submerged in 2 ml of RNAlater (Ambion, Austin, TX, USA). Only biopsy specimens showing a homogeneous histopathology of the testis parenchyma and samples yielding high-quality total RNA preparation were included in the analysis. Testicular histology was classified using a modification of the classical scoring procedure employed by Johnsen [28] as: (1) biopsies obtained from patients with post-testicular obstructions showing complete spermatogenesis, i.e. all stages represented and >20 late spermatids (also referred as "testicular spermatozoa") per seminiferous tubule (score count 10); (2) biopsies showing severe HS in all seminiferous tubules (score count 8); (3) biopsies from patients with SCOS (score count 2).

### Microarray analysis

Testis samples were homogenized using an hand glass potter, and total testis RNA extracted using the *SVtotal RNA Izolation System *kit (Promega, Madison, WI, USA). The purity and quantity of RNA was assessed using the Agilent 8453 Spectrophotometer (Agilent, Santa Clara, CA, USA). RNA quality was determined by both evaluation of the integrity of rRNA bands using agarose electrophoresis, and absorbtion readings at 260 and 280 nm. Extracted RNA was linearly amplified using the *Amino Allyl MessageAmp™ II aRNA Amplification Kit *(Ambion, Austin, TX, USA).

Five to ten ug of amplified aRNA was fluorescently labelled with Cy3-Cy5 cyanins and then hybridizated on high-density array (Microcribi Padova, Italy). The Human Array Ready Oligo Set Version 2 contains array able 70mers representing 21,329 well-characterized human genes in two replicates from the UniGene Database. This database is located at the National Center forBiotechnology Information (see http://www.ncbi.nlm.nih.gov/UniGene/). The UniGene database automatically clusters all human GenBank sequences into a non-redundant set of genes. Each cluster in the UniGene Database represents one unique gene. A cluster may contain manysequences, but one representative sequence has been selected based on the longest region of high-quality sequence data. All pathological testis RNAs were hybridized against normal testis RNA samples (patients with obstructive azoospermia and normal spermatogenesis) while the three samples with normal spermatogenesis were hybridized against an home-made RNA universal reference (brain, liver, muscle and lung), to generate a profile of genes specifically expressed in normal testis, for a total of 26 experiments (tab. 1). After hybridization, Cy3-Cy5 fluorescent signals were captured by a Confocal Laser Scanner "ScanArray Express" (Packard BioScience) and analysed using the software "ScanArray Express - MicroArray Analysis System" version *3.0 *(Perkin Elmer). Raw data of the performed experiments have been recorded in the GEO public database (accession number: GSE14310).

The values of the median signal intensity from each spot were subtracted from the local median background intensity. For each slide after local background subtraction, a LOWESS algorithm was used for row data normalization to evaluate signal to noise ratio and generate log ratios of sample vs. reference signal. Only spots showing a signal to noise ratio of at least 1.7 were included in the analysis.

Analysis of data obtained by microarray experiments was carried out by means of hierarchical gene clustering [29] using Cluster 3.0 (open source 2006) and TreeView (Stanford University Labs) software. In order to include in clustering analysis only well measured transcripts, we selected spots with a present call (identified transcripts with measurable expression) in at least 80% of experiments and being > 1.7 fold up- or down regulated in at least 8 experiments. Identified clusters were then analyzed by the Ingenuity Pathways Analysis (IPA) software (Ingenuity Systems, Redwood City, CA), in order to classify genes based on their biological functions and disclose the functional networks connecting specific genes.

### Web Resources

Microarray data are deposited in the GEO public database and are accessible without restriction at the following URL: http://www.ncbi.nlm.nih.gov/geo/query/acc.cgi?token=bhqhdyekysokofq&acc=GSE14310 (accession number: GSE14310).

## Competing interests

The authors declare that they have no competing interests.

## Authors' contributions

Study design VG, AF, CF, LS; Patients selection: AF, AG, CF, GP; Gene expression analysis: VG, FR, APS; RT-PCR analysis: FR, IA; Manuscript writing: VG, AF, GP, LS. All authors read and approved the final manuscript.

## Supplementary Material

Additional file 1 **List of transcripts (along with their relative accession numbers), that are found downregulated in the cluster of patients with AZFc deletion**. Click here for file

Additional file 2 **List of transcripts (along with the relative accession number) that are found upregulated in the cluster of patients with AZFc deletion**. Click here for file

Additional file 3 **Semiquantitative RT-PCR analysis of 6 major genes downregulated in pathological testis.** The housekeeping gene (GADPH: NP_002037.2) was used as control. 34 and 35 indicate the number of PCR cycles. Click here for file
