# Supplementary material for: Testis transcriptome analysis in male infertility: new insight on the pathogenesis of oligo-azoospermia in cases with and without AZFc microdeletion
Source: BMC Genomics. 2010 Jun 24;11:401. doi: 10.1186/1471-2164-11-401 (PMC2996929; doi:10.1186/1471-2164-11-401)

Fig.1: Semiquantitative RT-PCR analysis for 6 major genes down-regulated in pathological testis . The housekeeping gene (GADPH : NP_002037.2) was used as control. 34 and 35 indicate PCR cycle number.


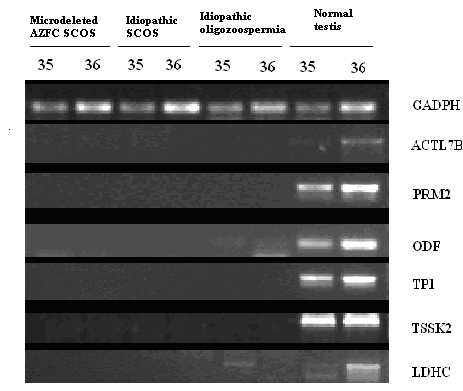

Supplement: Additional file 3 — Semiquantitative RT-PCR analysis of 6 major genes downregulated in pathological testis. The housekeeping gene (GADPH: NP_002037.2) was used as control. 34 and 35 indicate the number of PCR cycles. [file 1471-2164-11-401-S3.DOC]
